# Dendritic Cell Subset Distributions in the Aorta in Healthy and Atherosclerotic Mice

**DOI:** 10.1371/journal.pone.0088452

**Published:** 2014-02-14

**Authors:** Martin Busch, Thilo C. Westhofen, Miriam Koch, Manfred B. Lutz, Alma Zernecke

**Affiliations:** 1 Rudolf Virchow-Center; University of Würzburg, Würzburg, Germany; 2 Institute of Clinical Biochemistry and Pathobiocchemistry, University Hospital Würzburg, Würzburg, Germany; 3 Institute of Virology and Immunobiology, University of Würzburg, Würzburg, Germany; 4 Department of Vascular Surgery, Klinikum rechts der Isar der Technischen Universität München, Munich, Germany; Istituto Superiore di Sanità, Italy

## Abstract

Dendritic cells (DCs) can be sub-divided into various subsets that play specialized roles in priming of adaptive immune responses. Atherosclerosis is regarded as a chronic inflammatory disease of the vessel wall and DCs can be found in non-inflamed and diseased arteries. We here performed a systematic analyses of DCs subsets during atherogenesis. Our data indicate that distinct DC subsets can be localized in the vessel wall. In C57BL/6 and low density lipoprotein receptor-deficient (*Ldlr* ^−/−^) mice, CD11c^+^ MHCII^+^ DCs could be discriminated into CD103^−^ CD11b^+^F4/80^+^, CD11b^+^F4/80^−^ and CD11b^−^F4/80^−^ DCs and CD103^+^ CD11b^−^F4/80^−^ DCs. Except for CD103^−^ CD11b^−^ F4/80^−^ DCs, these subsets expanded in high fat diet-fed *Ldlr* ^−/−^ mice. Signal-regulatory protein (Sirp)-α was detected on aortic macrophages, CD11b^+^ DCs, and partially on CD103^−^ CD11b^−^ F4/80^−^ but not on CD103^+^ DCs. Notably, in FMS-like tyrosine kinase 3-ligand-deficient (*Flt3l* ^−/−^) mice, a specific loss of CD103^+^ DCs but also CD103^−^ CD11b^+^ F4/80^−^ DCs was evidenced. Aortic CD103^+^ and CD11b^+^ F4/80^−^ CD103^−^ DCs may thus belong to conventional rather than monocyte-derived DCs, given their dependence on Flt3L-signalling. CD64, postulated to distinguish macrophages from DCs, could not be detected on DC subsets under physiological conditions, but appeared in a fraction of CD103^−^ CD11b^+^ F4/80^−^ and CD11b^+^ F4/80^+^ cells in atherosclerotic *Ldlr* ^−/−^ mice. The emergence of CD64 expression in atherosclerosis may indicate that CD11b^+^ F4/80^−^ DCs similar to CD11b^+^ F4/80^+^ DCs are at least in part derived from immigrated monocytes during atherosclerotic lesion formation. Our data advance our knowledge about the presence of distinct DC subsets and their accumulation characteristics in atherosclerosis, and may help to assist in future studies aiming at specific DC-based therapeutic strategies for the treatment of chronic vascular inflammation.

## Introduction

Atherosclerosis is regarded as a chronic inflammatory disease of the vessel wall [1]. Both macrophages and dendritic cells (DCs) can be found in non-inflamed steady-state aortae and accumulate in atherosclerotic lesions [1]–[6]. While DCs in the intima are important in initial lesion formation by uptake of lipids and their transformation into foam-cell-like cells [7], evidence is accumulating that DCs are also critical in shaping adaptive immune responses in atherosclerosis [1], [5], [6], [8]–[10]. For instance, two DC subsets with distinct functions in controlling regulatory T cell homeoastasis and atherosclerotic lesion formation have been revealed [1], [10].

DCs can be sub-divided into various subsets that play specialized roles in priming of adaptive immune responses [11]. However, although CD11c is commonly accepted as a pan-DC marker, a clear identification of DCs is still limited by the lack of unambiguous surface markers. In particular, the discrimination of DCs from macrophages that share many markers and functions remains challenging. Recently, aortic CD11c^+^MHCII^+^ DCs were discriminated from CD11c^−^ MHCII^+^ macrophages by their low phagocytic activity but strong immune stimulatory capacities, and subdivided into CD11b^+^ F4/80^−^, CD11b^+^ F4/80^+^ as well as CD103^+^ subsets [10]. A systematic analysis of the presence of these subsets and their accumulation in the course of lesion development, however, has not yet been performed. Although it remains to be determined if these cell subsets denominated as DCs and macrophages represent true lineage subsets with distinct transcriptional profiles, we here have adopted and refined the gating strategy applied by Choi *et al*. and others and used the term DCs when describing cells that express CD11c, and macrophages for CD11c^−^ cells [6], [10], [12], [13]; we have characterized and quantified these cell subsets in C57BL/6 mice and atherosclerosis-prone mice fed a high fat diet. Furthermore, we applied novel surface markers to characterize these cell subsets during atherogenesis.

## Materials and Methods

### Mouse Models

CD11c-YFP reporter mice [14] were provided by M. Nussenzweig (Rockefeller University, New York, New York, USA). Apolipoprotein E-deficient (*ApoE* ^−/−^) mice, low-density-lipoprotein-receptor-deficient (*Ldlr* ^−/−^) mice and C57BL/6J mice were obtained from the Jackson laboratory. Fms-related tyrosine kinase 3 ligand (*Flt3l* ^−/−^) mice were obtained from Taconic. CD11c-YFP mice were crossed with *ApoE* ^−/−^ mice. Female mice were kept on a normal chow, or placed on a Western diet at 10–12 weeks of age for 6 or 12 weeks. Experiments were approved by local authorities and complied with German animal protection law. This study was carried out in accordance with the with German animal protection law, approved by the Regierung von Unterfranken, Würzburg, Germany (permit number 44/10).

### Enzymatic Tissue Digestion and Flow Cytometry

For FACS analysis, aortae were flushed with PBS, excised, carefully cleaned of all perivascular fat, and enzymatically digested with liberase TL (Roche) for 30 minutes at 37°C in RPMI-1640 (LifeTechnologies). Fc receptors were blocked with CD16/CD32 (clone 93) antibodies (eBioscience) in PBS supplemented with 2% mouse serum, 2% rabbit serum and 0.2% BSA prior to fluorescent labeling. Staining for flow cytometric analysis included fluorescently labeled antibodies against CD11c (PE-Cy7/AlexaFluor 488, clone N418), CD11b (AmCyan, clone M170), NK1.1 (PE-Cy7, clone PK136), CD103 (PE/APC, clone 2E7), SIRPα (FITC, clone P84), MHCII (eFlour450, clone M5/114.15.2), TCR-ß (PerCP-Cy5.5, clone H57–597), F4/80 (APC/PacBlue, clone BM8), PDCA (PE, clone eBio927) (eBioscience), CD19 (PerCP-Cy5.5, clone 1D3), CD45 (APC-Cy7, clone 30-F11), MHCII (FITC, clone 2G9) (BD Biosciences) and CD64 (APC, clone X54–5/7.1) (BioLegends) in HBSS with 0.3 mM EDTA and 0.1% BSA. Samples were analyzed using a FACSCanto II (BD Biosciences) and FlowJo software (Tree Star).

### Tissue Sections and Immunofluorescence Staining

The heart and aortae were perfused with 4% paraformaldehyde (PFA) in PBS, embedded in Tissue Tek and processed in 5 µm serial cryosections. Slides were mounted and nuclei were counterstained with 4′,6-Diamidino-2-phenylindole (DAPI, Vector laboratories). CD11c, F4/80 or CD68 staining was performed on cryosections of the aortic root using biotin-labeled anti-CD11c (Biolegend, clone N418), anti-CD68 (AbD Serotec, clone FA-11) or anti-F4/80 (AbD Serotec, clone CI:A3-1) antibodies, detected by Alexa-Fluor-555-Streptavidin (Molecular probes, Life Technologies), Alexa-Fluor-488 anti-rat IgG (Molecular probes, Life Technologies) or biotinylated anti-rat antibody and Alexa-Fluor-488-Streptavidin (Molecular probes, Life Technologies) respectively. Images were recorded using a Leica DMLB fluorescence microscope.

### Statistics

Data represent mean±SEM and were analyzed by Student’s t-test, ANOVA with Dunnett’s multiple comparison test (Prism 5.02 software, GraphPad), as appropriate. Differences where *P*<0.05 were considered to be statistically significant.

## Results

### Localization of CD11c^+^ Cells

**Figure 1:**
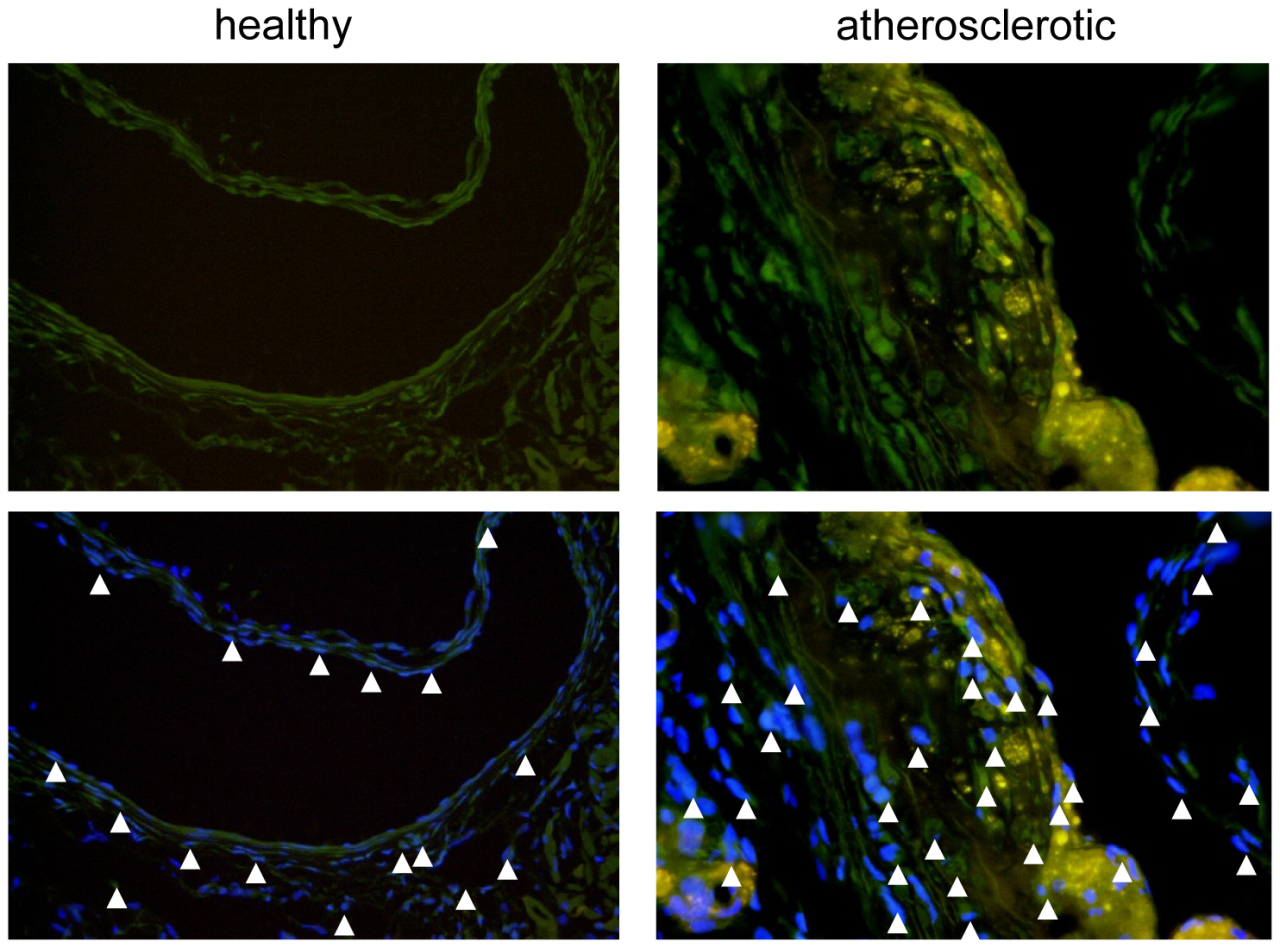
Localization of CD11c^+^ cells in the aortic root. (A) Aortic root sections of healthy and atherosclerotic *apoE* ^−/−^CD11c-YFP mice (arrow heads indicate CD11c^+^ cells, green). Nuclei are counterstained with DAPI (blue; scale bars, 50 µm).

Employing CD11c-YFP reporter mice, we used immunofluorescence microscopy to localize CD11c^+^ cells in chow-fed healthy as well as atherosclerotic apolipoprotein E-deficient (*apoE*)^−/−^ mice. CD11c^+^ cells could be detected in the intima, adventitia, and within the valves in the aortic sinus in CD11c-YFP reporter mice, in line with previous findings [4], [6], [15], [16] (**Figure 1**). In atherosclerotic CD11c-YFP *apoE* ^−/−^ mice, numerous CD11c^+^ cells were detectable throughout the intima with a preference to luminal plaque regions and the adventitia in the aortic sinus (**Figure 1**); in the aorta, in particular in regions of the aortic arch but also in thoracic and abdominal segments, and in the innominate artery, we similarly observed CD11c^+^ cells within plaques, whereas CD11c^+^ cells could only rarely be found in the adventitia in these regions (**Figure S1**, and not shown).

### Identification and Quantification of Distinct Aortic DC Subsets

**Figure 2:**
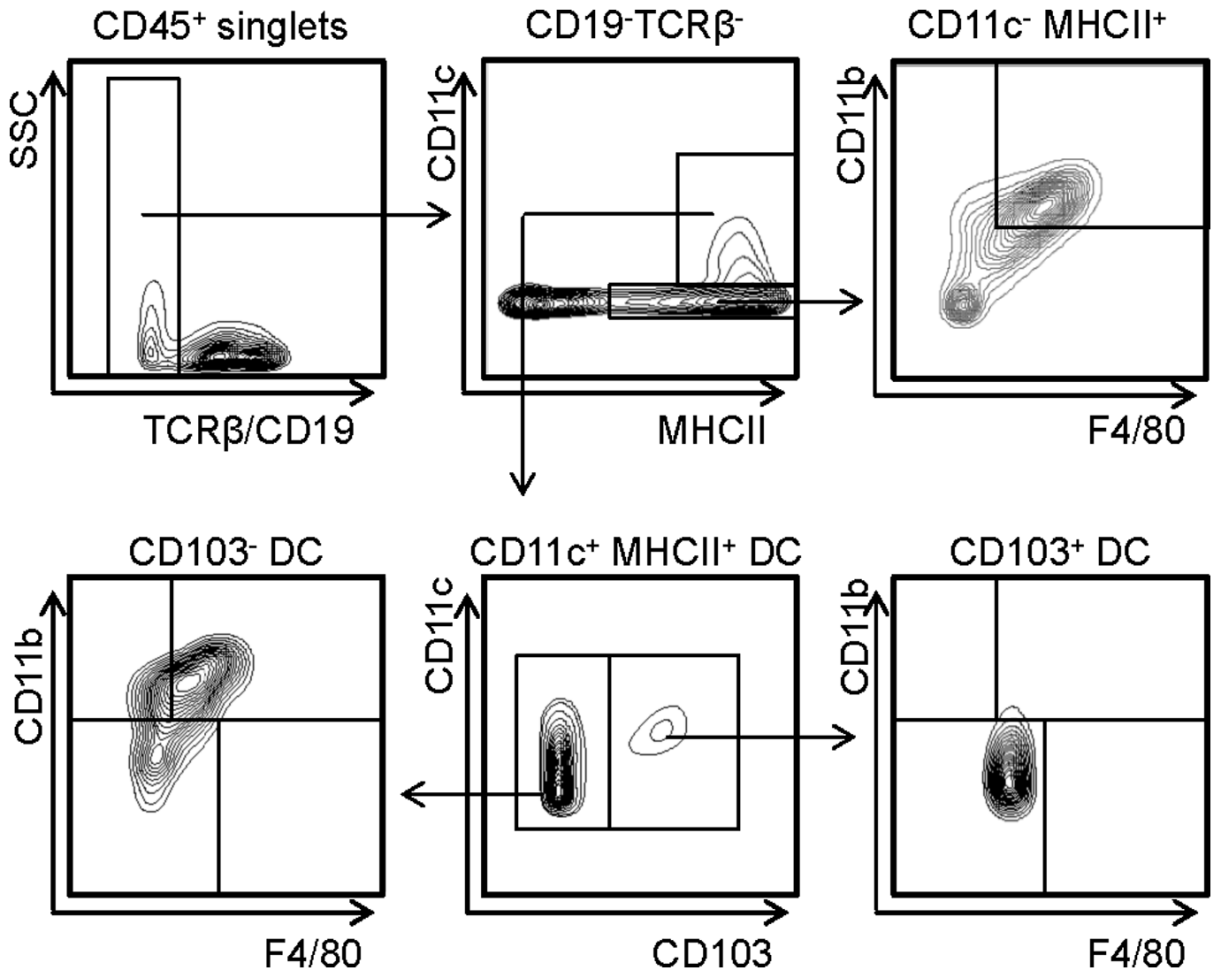
Identification of DC subsets in the aorta. Representative FACS plots for identification of DC subsets in healthy, chow-fed *Ldlr* ^−/−^ mice. After exclusion of TCRβ^+^/CD19^+^ T and B cells, CD11c^+^ MHCII^+^ DCs were further subdivided into CD103^+^ and CD103^−^ DCs. CD103^+^ DCs do not express CD11b or F4/80 while CD103^−^ DCs were further subdivided into CD11b^+^ F4/80^−^, CD11b^+^ F4/80^+^ and CD11b^−^F4/80^−^ DCs. Macrophages were defined as CD11c^−^ MHCII^+^ CD11b^+^ F4/80^+^. Representative contour plots from 6–9 mice per group are shown.

To phenotypically characterize and quantify DC subsets in healthy and atherosclerotic arteries, enzymatically digested aortae were subjected to multicolour flow cytometry. After exclusion of CD19^+^ and TCRβ^+^ B and T cells, respectively, DCs were identified as CD11c^+^ MHCII^+^ cells within CD45^+^ leukocytes [6], [10], [12], [13] in C57BL/6 mice (not shown) and chow-fed low density lipoprotein receptor-deficient (*Ldlr* ^−/−^) mice (**Figure 2**). Whereas the majority of aortic CD11c^+^ MHCII^+^ DCs showed no staining for E-cadherin-ligand CD103, a small CD103^+^ DC population could be confined. This subset displayed no expression of CD11b or F4/80 (**Figure 2**), in line with previous data [10]. In contrast, the CD103^−^ fraction of aortic DCs could further be subdivided into a large population of CD11c^+^ CD11b^+^ F4/80^+^ DCs and a less abundant CD11c^+^ CD11b^+^ F4/80^−^ subset, as described previously [10], but also CD11c^+^ CD11b^−^ F4/80^−^ cells (**Figure 2**). Within the subset of CD11c^+^ CD11b^−^ F4/80^−^ cells we were able to identify sporadic CD11c^lo^ CD11b^−^ PDCA^+^ cells, in line with the presence of few plasmacytoid DCs (pDCs) [17], [18]. Due to their low numbers (125±42 pDCs per aorta, amounting to approximately 1.6% of CD11c^+^ MHCII^+^ DCs), we refrained from further studying this DC subset in subsequent analyses in this study. All DC subsets were negative for expression of the NK/NKT cell marker NK1.1 (data not shown). In line with previous gating strategies [10], CD11c^−^ MHCII^+^ cells that co-stained for CD11b and F4/80 were denominated as macrophages (**Figure 2**).

**Figure 3:**
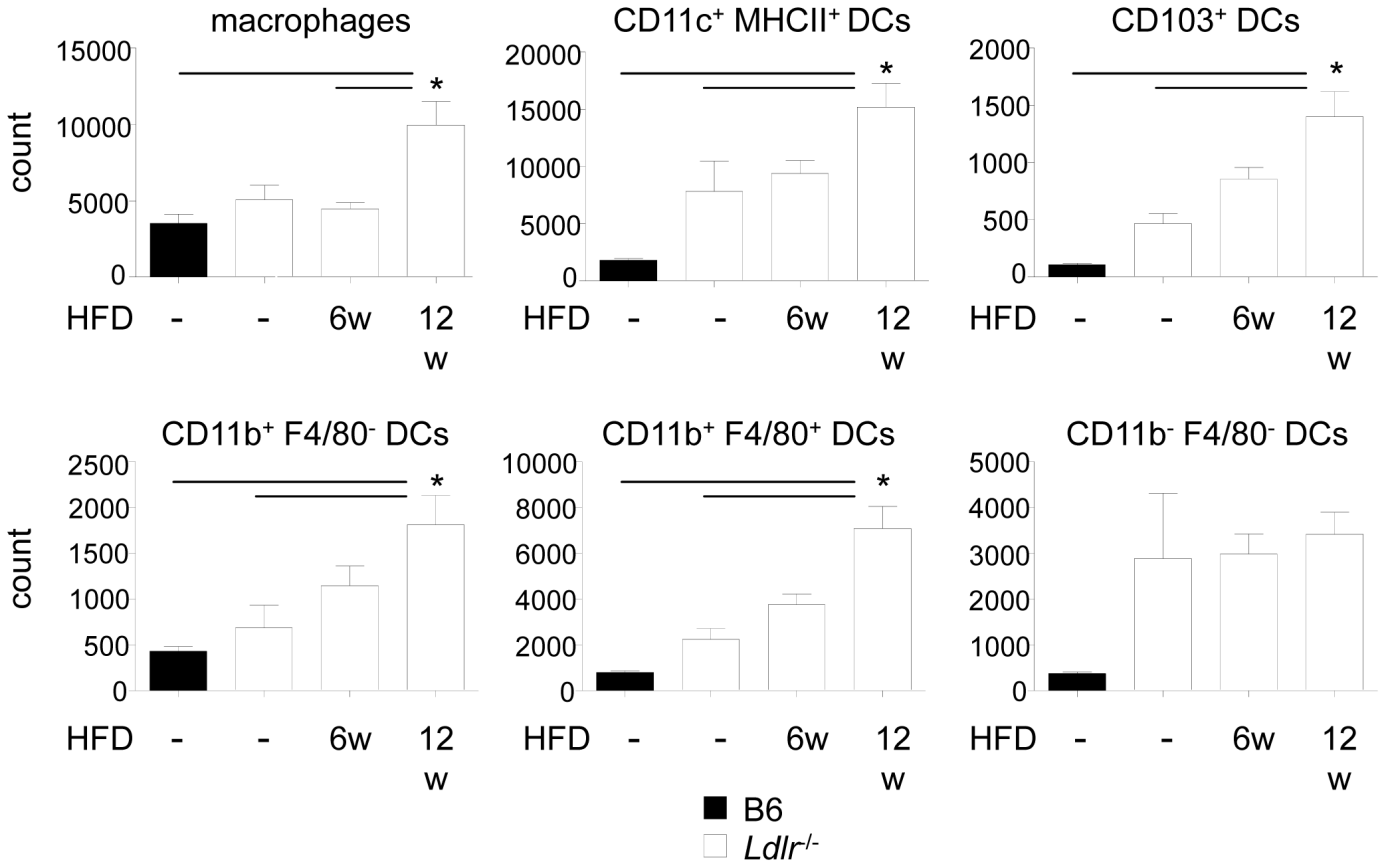
Quantification of aortic DC subsets. Aortic DC and macrophage numbers were quantified in the aorta of C57BL/6J and *Ldlr* ^−/−^ mice fed a normal chow, or in *Ldlr* ^−/−^ mice after 6 or 12 weeks of high fat diet-feeding by flow cytometry (n = 6–9 mice per group; data were obtained by pooling 2–3 aortae per experiment and then calculating the average number of cells per mouse and aorta, and represent 6–9 independent experiments). Data represent mean±SEM. *P<0.05.

To assess the kinetics of the accumulation of these cell subpopulations during atherogenesis, we compared aortic digests of C57BL/6 mice with *Ldlr* ^−/−^ mice fed a normal chow and *Ldlr* ^−/−^ mice fed a high fat diet (HFD) for 6 and 12 weeks. In line with the presence of these cell types in healthy mice [3], [4], [6], [16], numerous macrophages and also DCs could already be identified in C57BL/6 mice (**Figure 3**). *Ldlr* ^−/−^ mice on normal chow displayed a non-significant trend towards elevated numbers of DCs when compared to C57BL/6 mice. With the duration of diet, DC numbers significantly increased in *Ldlr* ^−/−^ mice (**Figure 3**). While the subpopulations of CD11c^+^ CD103^+^ and CD11c^+^ CD11b^+^ F4/80^+^ DCs showed the most marked expansion among all DC subsets, the smaller population of CD11c^+^ CD11b^+^ F4/80^−^ DCs showed a less dramatic increase. CD11c^+^ CD11b^−^ F4/80^−^ DCs did not contribute to the increase in DC numbers with diet. Analysis of the frequencies of distinct DCs subsets among all DCs showed a slight but non-significant expansion of CD11c^+^ CD103^+^ DCs, whereas relative numbers of CD11c^+^ CD11b^+^ F4/80^−^ DCs decreased in *Ldlr* ^−/−^ mice fed a HFD for 12 weeks compared to C57BL/6 mice; percentages among all other DC subsets showed no alterations in the course of atherosclerotic lesion development (**Figure S2**).

**Figure 4:**
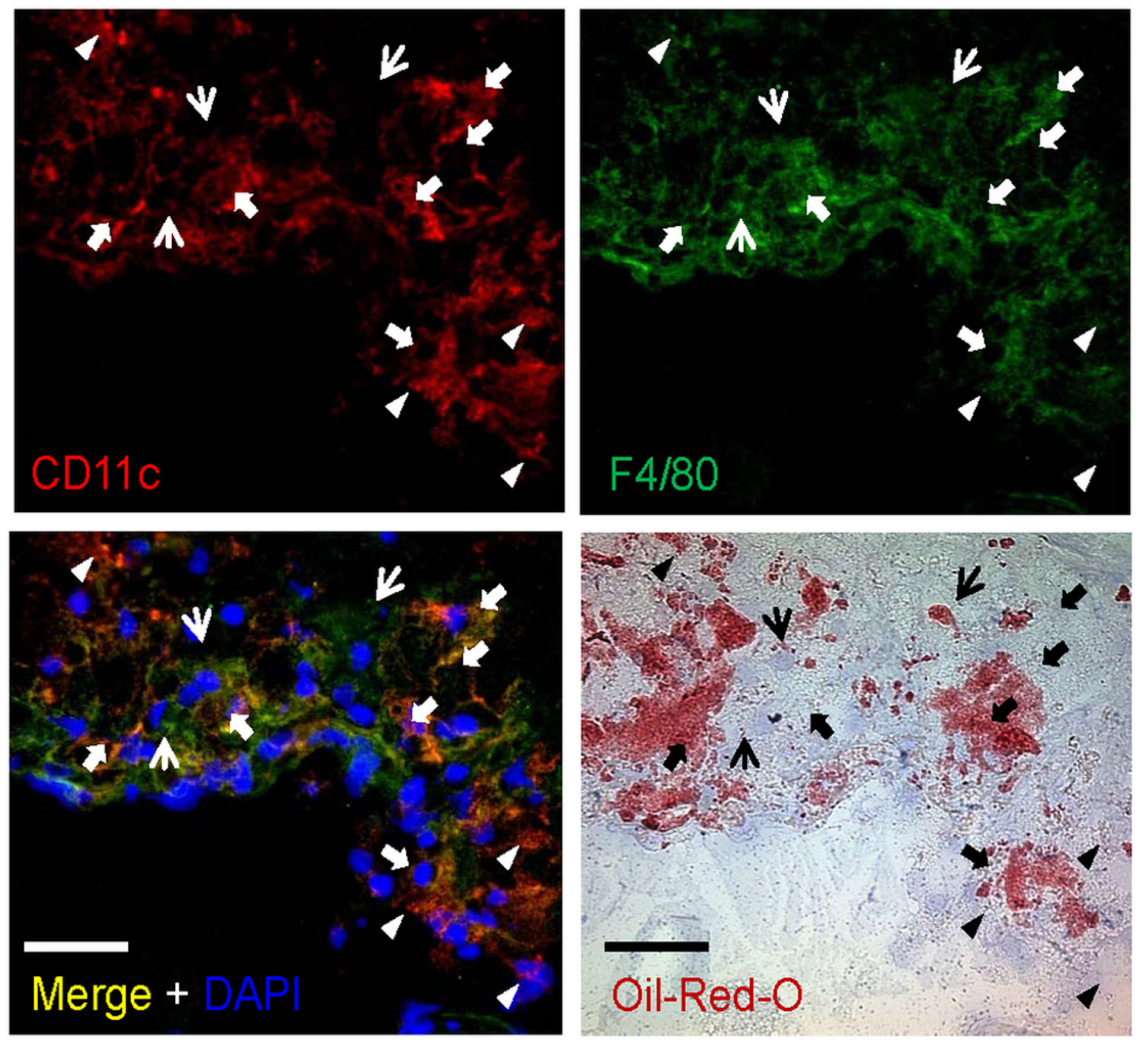
Characterization of aortic DC subsets. Representative co-immunofluorescence staining of aortic root sections of *Ldlr* ^−/−^ mice fed a high fat diet for 12 weeks, revealing cells showing staining for only CD11c (red, filled arrow heads) or F4/80^+^ (green, narrow arrows) as well as both CD11c and F4/80 (yellow, bold arrows). Nuclei are counterstained with DAPI (blue). Oil-red-O staining (red) for lipids in adjacent sections. Scale bars, 50 µm.

To further validate data obtained by flow cytometry, co-immunofluorescence staining for CD11c and F4/80, as well as CD11c and CD68 were performed in sections of the aortic root of *Ldlr* ^−/−^ mice fed a HFD for 12 weeks. As expected, we were able to identify cells positive for CD11c or F4/80 only, but also double positive CD11c^+^ F4/80^+^ cells, in line with the presence of CD11c^+^ F4/80^+^ and CD11c^+^F4/80^−^ DCs, as well as CD11c^−^ F4/80^+^ macrophages, furthermore supporting our findings from flow cytometry (**Figure 4**). Similar results were obtained for CD11c and CD68 (**Figure S3**). In line with published data that also CD11c^+^ cells can take on a foam cell-like appearance upon the engulfment of lipids similar to macrophages [6], [7], no clear further discrimination into different cell populations was possible based on staining for lipids by Oil-red-O.

### Characterization of Aortic DC Subsets

**Figure 5:**
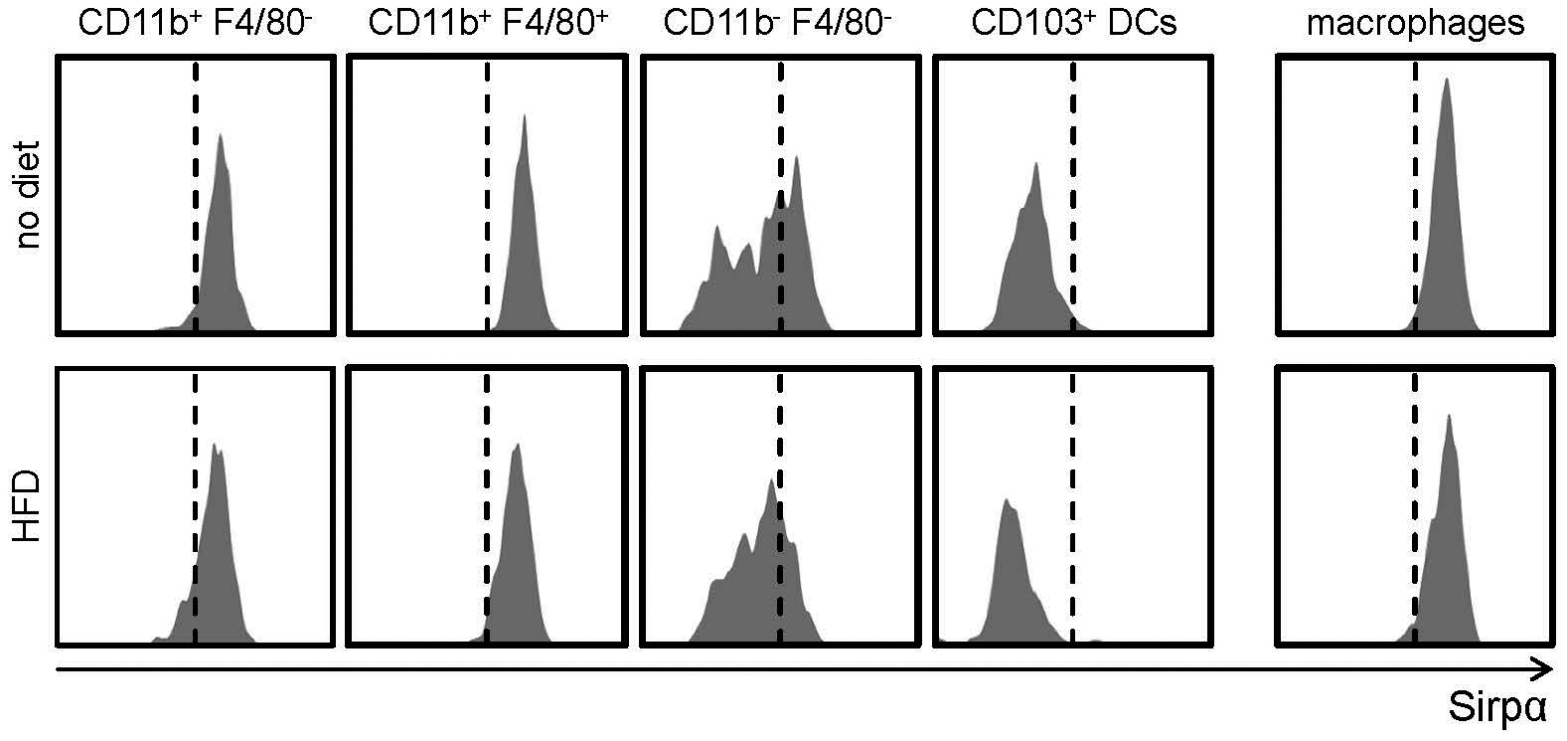
SIRPα expression on aortic DC subsets and macrophages. Representative histograms of the expression of SIRPα on DC subsets and macrophages in the aorta of *Ldlr^−/−^* mice fed a normal chow or after 12 weeks of high fat diet-feeding. Representative histograms from 6–9 mice per group are shown.

The signal-regulatory protein (Sirp)-α, a transmembrane receptor with functions in cell migration and phagocytosis, was recently described to be expressed on CD11b^+^ DCs and monocytes/macrophages [11], [19]–[21]. Its expression had not been investigated in aortic macrophages and DCs. We here demonstrate that CD11c^+^CD11b^+^F4/80^−^ and CD11c^+^CD11b^+^F4/80^+^ DCs express SIRPα, similarly to CD11c^−^ MHCII^+^ F4/80^+^ macrophages, whereas CD11c^+^CD11b^−^ F4/80^−^ DCs display intermediate levels; CD11c^+^CD103^+^ DCs do not express SIRPα (**Figure 5**). No differences in SIRPα expression were noted between chow-fed C57BL/6 and *Ldlr* ^−/−^ mice or HFD-fed *Ldlr* ^−/−^ mice (not shown, and **Figure 5**).

The discrimination of monocytes/macrophages from DCs remains controversial. In the aorta, Choi *et al*. distinguished these cell types by functional criteria, i.e. phagocytic *versus* immune stimulatory capacities [10]. Moreover, CD11c^−^ F4/80^+^ CD11b^+^ macrophages were shown to depend on hematopoietin M-CSF, in line with a monocytic origin, whereas DCs were of two types, with CD11b^+^ F4/80^+^ DCs being M-CSF but CD103^+^ DCs being FMS-like tyrosine kinase 3 (Flt3)-dependent, as revealed by analysing *op/op* and *Flt3* ^−/−^ mice on normal chow, respectively [10]. We analyzed aortic DC distributions in *Flt3l* ^−/−^ mice. Similar to findings in *Flt3* ^−/−^ mice [10], we observed a trend towards reduced total CD11c^+^MHCII^+^ DC counts in *Flt3l* ^−/−^ compared to C57BL/6 mice (999±287 *vs*. 1827±137 cells per aorta, respectively; n = 3 independent experiments; n.s) but no changes in CD11c^−^CD11b^+^F4/80^+^ macrophages (2929.0.2±763.1 *vs*. 3517.0±605.3 cell, n.s). The analysis of individual DC populations revealed no alterations in CD11c^+^CD11b^+^F4/80^+^ DCs (512.2±143.2 *vs*. 797.2±63.1 cells, n.s.) and CD11c^+^CD11b^−^ F4/80^−^ DCs (382.3±95.9 *vs*. 377.3±36.8 cells, n.s.) in *Flt3l* ^−/−^ compared to C57BL/6 mice. Similar to *Flt3* ^−/−^ mice, we evidenced a complete absence of CD11c^+^CD103^+^ DCs in *Flt3l* ^−/−^ compared to C57BL/6 mice. Notably, we in addition evidenced an almost complete loss of CD11c^+^CD11b^+^ F4/80^−^ DCs in *Flt3l* ^−/−^ mice (**Figure S4**). These findings demonstrate that not only aortic CD103^+^ DCs but also CD11c^+^CD11b^+^ F4/80^−^ DCs depend on Flt3/Flt3L signaling under physiological conditions, whereas CD11c^+^CD11b^+^F4/80^+^ and CD11b^−^F4/80^−^ DCs, similar to macrophages, accumulate Flt3L-independently in the aorta in healthy mice.

**Figure 6:**
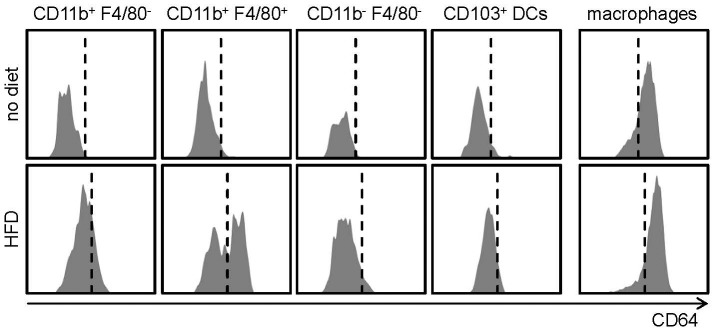
CD64 expression on aortic DC subsets and macrophages. Representative histograms of CD64 expression on aortic DC subsets and macrophages. Representative histograms from 6–9 mice per group are shown.

Recent gene-expression studies (Immunological Genome project) have revealed the high-affinity Fcγ-receptor-I CD64 as a promising marker to distinguish macrophages from CD11b^−^ DCs [22]. Furthermore, CD64 was shown to be expressed on monocyte-derived DCs but not conventional DCs [23], [24]. We therefore tested the expression of CD64 on macrophages and on the identified distinct DC subsets. While CD11c^−^ CD11b^+^ F4/80^+^ macrophages showed uniform staining for CD64 (serving as a positive control), none of the CD11c^+^ MHCII^+^ DC subsets displayed any surface expression of CD64 in C57BL/6 or *Ldlr* ^−/−^ mice on normal chow (not shown, **Figure 6**). These findings could suggest that aortic CD11c^+^ subsets are conventional DCs rather than monocyte-derived DCs under physiological conditions. In contrast, when analyzing atherosclerotic *Ldlr* ^−/−^ mice fed a HFD for 12 weeks, a small fraction of CD11c^+^ CD11b^+^ F4/80^−^ DCs, and about half of all CD11c^+^ CD11b^+^ F4/80^+^ DCs stained positive for CD64, while CD11c^+^ CD11b^−^ F4/80^−^ DCs and CD11c^+^ CD103^+^ DCs remained negative (**Figure 6**). Among all DCs, about 21% of cells stained positive for CD64 under atherogenic conditions (not shown). All macrophages remained CD64^+^ also in atherosclerotic mice (**Figure 6**). These data suggest that predominantly CD11c^+^ CD11b^+^ F4/80^+^ aortic DC subsets may be of monocytic origin in atherosclerosis.

## Discussion

Extending previous studies [6], [10], [16] we here further refine the characterization of DCs in the aorta. After segregation of CD11c^−^ MHCII^+^ cells, denominated as macrophages [10], [12], [13], we further discriminated CD11c^+^ MHCII^+^ ‘DCs’ into a distinct CD103^+^ population, and CD103^−^ DCs that could further be subdivided into CD11b^−^ F4/80^+^ and CD11b^+^ F4/80^+^ DCs, similar to DC populations defined by gating strategies proposed by others [6], [10]. In addition we here were able describe a CD11b^−^ F4/80^−^ DC population. While it was demonstrated that all CD11b^−^ F4/80^−^ DCs express CD103 [10], we identified two distinct CD11b^−^ F4/80^−^ DC subsets, namely CD11b^−^ F4/80^−^ CD103^+^ DCs (as previously described by Choi *et al*.), but also CD11b^−^ F4/80^−^ CD103^−^ DCs. Koltsova *et al*. have previously referred to a subset of CD11c^+^CD11b^−^ DCs in the aorta, which may have overlapped with both CD11b^−^F4/80^−^ CD103^+^ and CD11b^−^F4/80^−^ CD103^−^ DCs [6]. These aortic DCs were present in healthy and diseased aortae (independent of atherosclerotic lesion formation). While we excluded staining for CD3, CD19, CD103, NK1.1, F4/80, CD11b and CD64, this CD11c^+^ MHCII^+^ DC subset remains to be characterized in more detail in future studies.

In line with its expression on monocytes/macrophages and CD11b^+^ DCs [11], [19], [20], we here for the time revealed SIRPα expression primarily on aortic macrophages, CD11b^+^ F4/80^+^ and CD11b^+^ F4/80^−^ DCs, whereas the newly identified subset of CD11b^−^ F4/80^−^ CD103^−^ DCs showed partial staining for SIRPα.

Consistent with previous findings, substantially more macrophages than DCs could be detected in non-diseased, healthy vessels, which may primarily localize to the adventitia. In contrast, DCs have been ascribed to accumulate in the intima and in regions predisposed to atherosclerosis in non-diseased mice [3], [4], [10]. Although it was suggested that intimal DCs may originate from circulating monocytes [4], previous studies lacked DC subset-specific analyses, and global gene expression analyses, for example from intimal regions, demonstrated expression of DC and macrophage markers [4]. Choi *et al*. showed that the subset of CD11b^+^ F4/80^+^ DCs (but not CD11b^+^ F4/80^−^ DCs), similarly to macrophages, strongly depends on M-CSF and expresses CX_3_CR1 and CD14 in healthy mice, consistent with a potential monocytic origin. In contrast, only CD103^+^ DCs were identified as true DCs requiring Flt3 for their accumulation in the aorta [10].

None of the CD11c^+^ MHCII^+^ DCs displayed any surface expression of CD64 in healthy mice, postulated as a potentially powerful marker to distinguish macrophages from DCs [22] and to differentiate monocyte-derived DCs from conventional (CD11b^+^) DCs [23]. Moreover, both CD11c^+^ CD103^+^ DCs and CD11c^+^ CD103^−^ CD11b^+^ F4/80^−^ DCs were Flt3L-dependend and lacked surface expression of CD64. This would suggest not only aortic CD103^+^ [10] but also CD103^−^ CD11b^+^ F4/80^−^ DCs to belong to conventional rather than monocyte-derived DCs and to differentiate from a designated DC precursor in the aorta under physiological conditions. This would also be supported by published data that show a reduction in both CD103^+^ and CD11b^+^ DCs in *Flt3* and *Flt3l*-deficient mice in other nonlymphoid tissue [25], [26], in accordance with a role of Flt3L in driving the differentiation of progenitor cells (but not monocytes) to the DC lineage [27] and Flt3-dependent common DC precursors (CDPs) and pre-DCs to give rise to both CD103^+^ and CD11b^+^ cDCs in the liver and kidney [25]. Likewise,CD64^−/lo^CD11b^+^ DCs were recently shown to develop in a Flt3L-dependent manner under steady state conditions in the skin [24]. CD11c^−^ macrophages and CD11b^+^ F4/80^+^ DCs were unaffected by the deficiency of Flt3L in our experiments under physiological conditions. The exact differentiation pathways of DCs, in particular of CD11c^+^ CD11b^+^F4/80^+^ and CD11c^+^ CD103^−^CD11b^−^F4/80^−^ DCs, in the aorta in healthy mice, however, remain to be defined.

Notably, in the skin CD64^lo/+^ CD11b^+^ DCs were shown to develop from circulating Ly-6C^hi^ monocytes and to display low to intermediate levels of CD64 [24], which may similarly apply to monocyte-derived DCs in the aorta in mice. The emergence of CD64^lo/+^ CD11b^+^ F4/80^−^ and CD11b^+^ F4/80^+^ DCs in the aorta in atherosclerosis may thus indicate that these subsets are in part derived from immigrated monocytes during atherosclerotic lesion formation. Although it may be possible that the marker CD64 does not assist in defining the origin of aortic DCs, we can rule out that lack of CD64 staining was due to technical problems, given the uniform staining of CD64 in macrophages in all experiments.

Notably, higher numbers of CD11c^+^ DCs than CD11c^−^ macrophages [10] were observed in advanced atherosclerotic plaques. Given that CD11c^+^MHCII^+^CD11b^+^ F4/80^+^ DCs represent the largest DC subpopulation, and that macrophages can express CD11c under inflammatory conditions [6], [28], it remains to be defined whether this discrimination into DCs *versus* macrophages in the aorta, based on the expression of CD11c, holds true in future studies, in particular, as these cell subsets share similar expression of F4/80 and CD11b. Together with a foam cell-like appearance of CD11c^+^ cells in the intima upon the initiation of a HFD [6], [7] and in atherosclerotic lesions of CD11c-YFP *apoE* ^−/−^ reporter mice in our study, a further functional characterization of the capacity of aortic CD11c^+^ and CD11c^−^ cells and of individual DC subsets (with regards to migration, phagocytosis and immune priming) is clearly warranted not only in healthy mice [10], but also under conditions of HFD to resolve this question. Unambiguous surface markers to discriminate these cell populations remain to be defined. Moreover, a decision on whether the gated DC populations represent true lineage subsets (as seen for CD4^+^ and CD8^+^ T cells), display different transcriptionally active states (as in Th1, Th2 and Tregs), or whether DC subsets just up- or down-regulate these surface markers (as also known for T cells or for macrophages during M1/M2 polarization) remains to be determined.

Our data enhance our knowledge about the presence of distinct DC subsets and their accumulation in atherosclerosis. The systematic survey of the phenotype and possible origin of aortic DCs are prerequisites for future studies aiming at defining specific DC-based therapeutic strategies for the treatment of chronic vascular inflammation.

## Supporting Information

Figure S1 **Localization of CD11c^+^ cells in different vascular beds.** Representative sections of the innominate artery and the aortic arch of atherosclerotic CD11c-YFP *ApoE^−/−^* reporter mice (arrow heads indicate CD11c^+^ cells, green). Nuclei are counterstained with DAPI (blue; scale bars, 50 µm).
(PDF)

Figure S2 **Aortic DC subset distribution.** DC subsets as percentages of total DCs in the aorta of Bl6 and *Ldlr^−/−^* mice fed a normal chow, or in *Ldlr^−/−^* mice after 6 or 12 weeks of high fat diet-feeding (6–9 mice per group). Data represent mean±SEM. *p<0.05.
(PDF)

Figure S3 **Characterization of aortic DC subsets.** Representative co-immunofluorescence staining of aortic root sections of *Ldlr* ^−/−^ mice fed a high fat diet for 12 weeks, revealing cells showing staining for only CD11c (red, filled arrow heads) or CD68^+^ (green, narrow arrows) as well as both CD11c and CD68 (yellow, bold arrows). Nuclei are counterstained with DAPI (blue). Oil-red-O staining (red) for lipids in adjacent sections. Scale bars, 50 µm.
(PDF)

Figure S4 **DCs in the Flt3L-deficient mice.** Representative FACS plots for identification of DC subsets in healthy *Flt3l* ^+/+^ and *Flt3l^−/−^* mice, fed a normal chow. After exclusion of TCRβ^+^/CD19^+^ T and B cells, macrophages were defined as CD11c^−^ MHCII^+^ CD11b^+^ F4/80^+^ (left panel), and CD11c^+^ MHCII^+^ DCs were further subdivided into CD103^+^ and CD103^−^ DCs (middle panel). CD103^−^ DCs were further subdivided into CD11b^+^ F4/80^−^, CD11b^+^ F4/80^+^ and CD11b^−^F4/80^−^ DCs (right panel).
(PDF)
