# Supplementary material for: Dendritic Cell Subset Distributions in the Aorta in Healthy and Atherosclerotic Mice
Source: PLoS One. 2014 Feb 14;9(2):e88452. doi: 10.1371/journal.pone.0088452 (PMC3925240; doi:10.1371/journal.pone.0088452)
Supplement: Figure S1 — Localization of CD11c+ cells in different vascular beds. Representative sections of the innominate artery and the aortic arch of atherosclerotic CD11c-YFP ApoE−/− reporter mice (arrow heads indicate CD11c+ cells, green). Nuclei are counterstained with DAPI (blue; scale bars, 50 µm). (PDF) [file pone.0088452.s001.pdf]

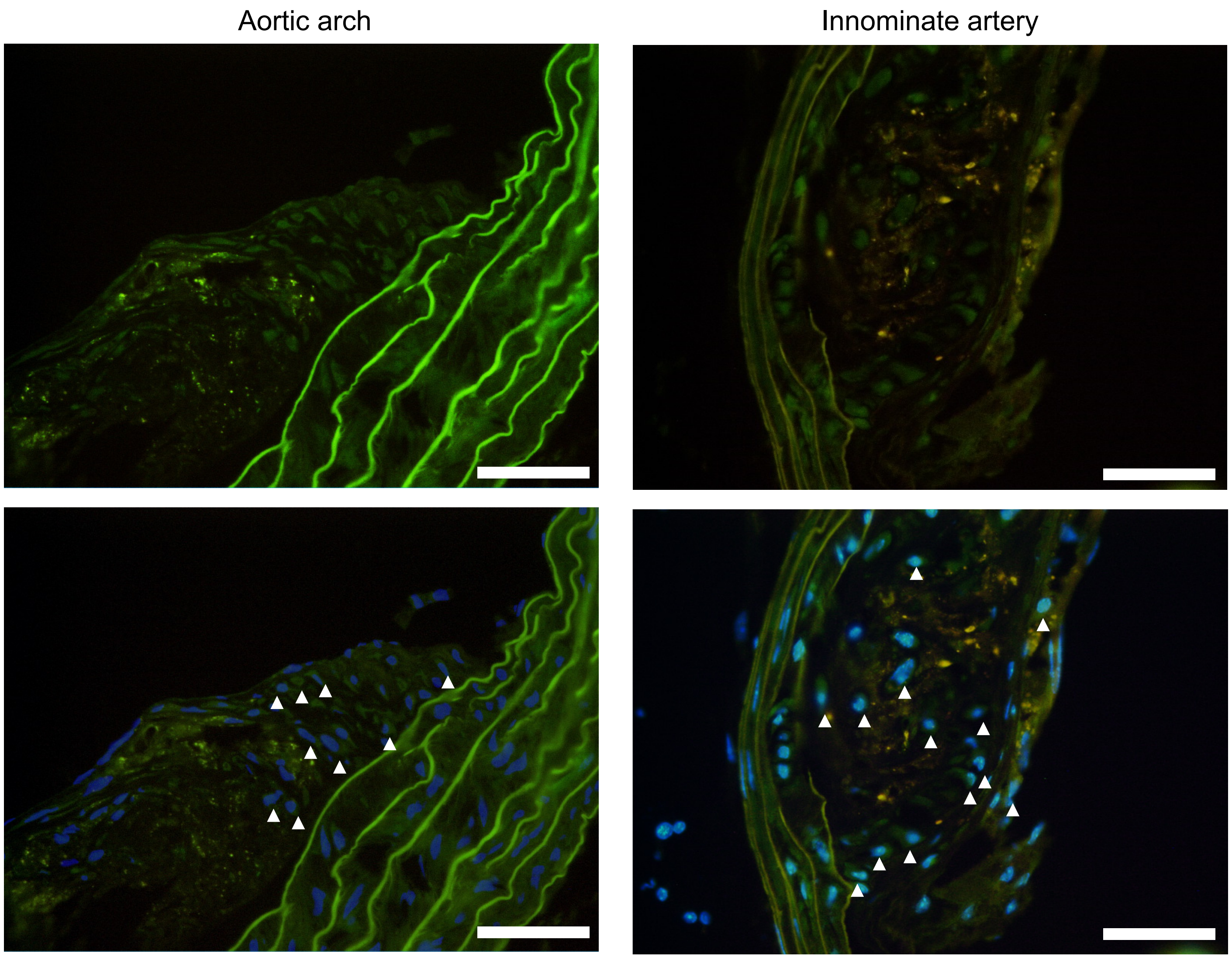

Supplemental Figure S1. Localization of CD11c^{+} cells in different vascular beds.

Representative sections of the innominate artery and the aortic arch of atherosclerotic CD11c-YFP *ApoE*$^{-/-}$ reporter mice (arrow heads indicate CD11c^{+} cells, green). Nuclei are counterstained with DAPI (blue; scale bars, 50µm).
