# Supplementary material for: Dendritic Cell Subset Distributions in the Aorta in Healthy and Atherosclerotic Mice
Source: PLoS One. 2014 Feb 14;9(2):e88452. doi: 10.1371/journal.pone.0088452 (PMC3925240; doi:10.1371/journal.pone.0088452)
Supplement: Figure S2 — Aortic DC subset distribution. DC subsets as percentages of total DCs in the aorta of Bl6 and Ldlr−/− mice fed a normal chow, or in Ldlr−/− mice after 6 or 12 weeks of high fat diet-feeding (6–9 mice per group). Data represent mean±SEM. *p<0.05. (PDF) [file pone.0088452.s002.pdf]

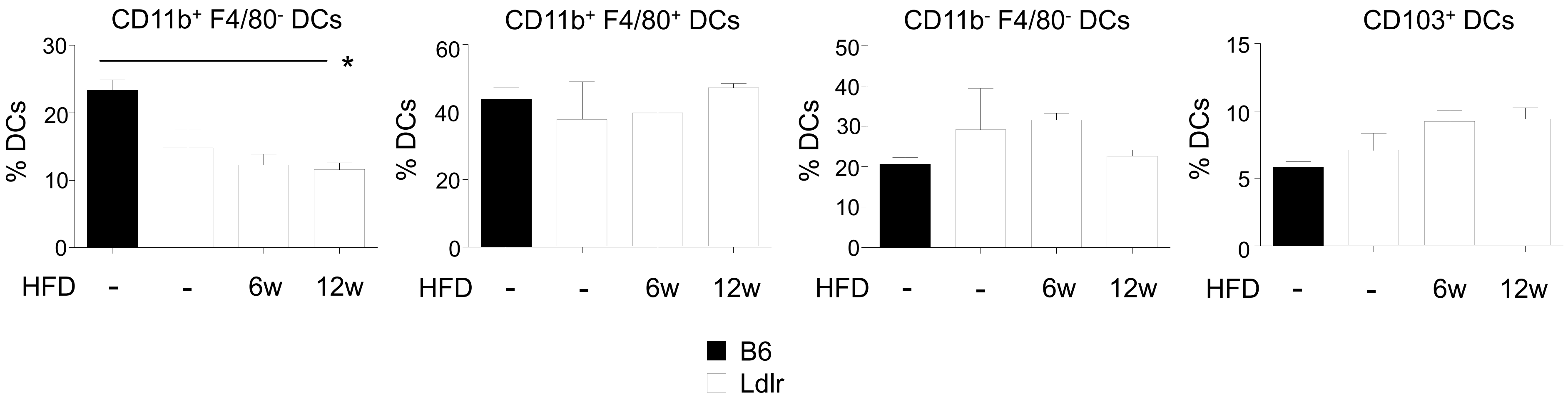

Supplemental Figure S2. Aortic DC subset distribution.

DC subsets as percentages of total DCs in the aorta of Bl6 and *Ldlr*$^{-/-}$ mice fed a normal chow, or in *Ldlr*$^{-/-}$ mice after 6 or 12 weeks of high fat diet-feeding (6-9 mice per group). Data represent mean±SEM. **p*<0.05.
