# Supplementary material for: Dendritic Cell Subset Distributions in the Aorta in Healthy and Atherosclerotic Mice
Source: PLoS One. 2014 Feb 14;9(2):e88452. doi: 10.1371/journal.pone.0088452 (PMC3925240; doi:10.1371/journal.pone.0088452)
Supplement: Figure S3 — Characterization of aortic DC subsets. Representative co-immunofluorescence staining of aortic root sections of Ldlr −/− mice fed a high fat diet for 12 weeks, revealing cells showing staining for only CD11c (red, filled arrow heads) or CD68+ (green, narrow arrows) as well as both CD11c and CD68 (yellow, bold arrows). Nuclei are counterstained with DAPI (blue). Oil-red-O staining (red) for lipids in adjacent sections. Scale bars, 50 µm. (PDF) [file pone.0088452.s003.pdf]

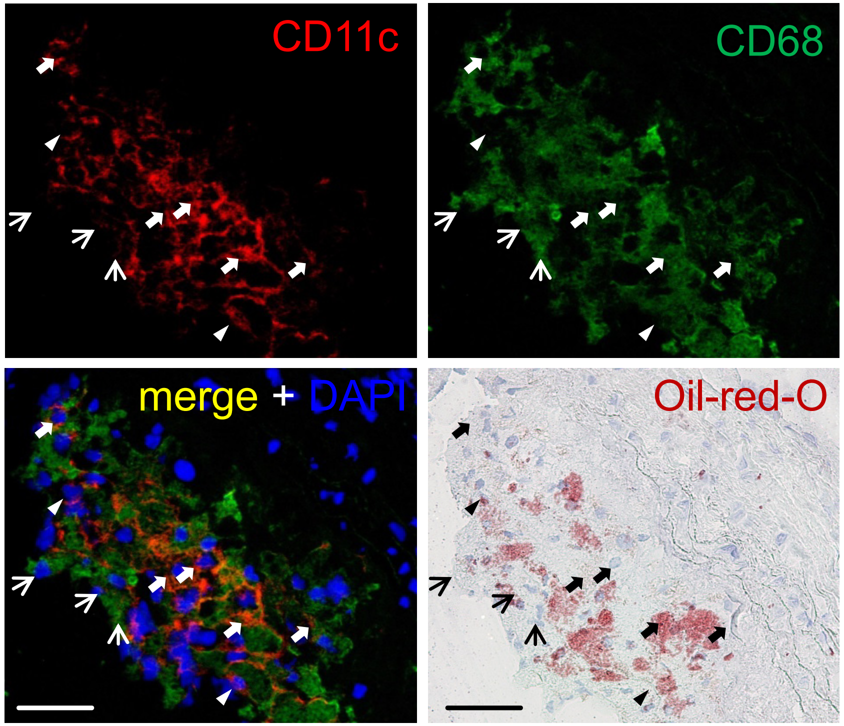


**Supplemental Figure S3. Characterization of aortic DC subsets.**

Representative co-immunofluorescence staining of aortic root sections of *Ldlr*$^{-/-}$ mice fed a high fat diet for 12 weeks, revealing cells showing staining for only CD11c (red, filled arrow heads) or CD68$^{+}$ (green, narrow arrows) as well as both CD11c and CD68 (yellow, bold arrows). Nuclei are counterstained with DAPI (blue). Oil-red-O staining (red) for lipids in adjacent sections. Scale bars, 50 µm.
