# Supplementary material for: Dendritic Cell Subset Distributions in the Aorta in Healthy and Atherosclerotic Mice
Source: PLoS One. 2014 Feb 14;9(2):e88452. doi: 10.1371/journal.pone.0088452 (PMC3925240; doi:10.1371/journal.pone.0088452)
Supplement: Figure S4 — DCs in the Flt3L-deficient mice. Representative FACS plots for identification of DC subsets in healthy Flt3l +/+ and Flt3l−/− mice, fed a normal chow. After exclusion of TCRβ+/CD19+ T and B cells, macrophages were defined as CD11c− MHCII+ CD11b+ F4/80+ (left panel), and CD11c+ MHCII+ DCs were further subdivided into CD103+ and CD103− DCs (middle panel). CD103− DCs were further subdivided into CD11b+ F4/80−, CD11b+ F4/80+ and CD11b−F4/80− DCs (right panel). (PDF) [file pone.0088452.s004.pdf]

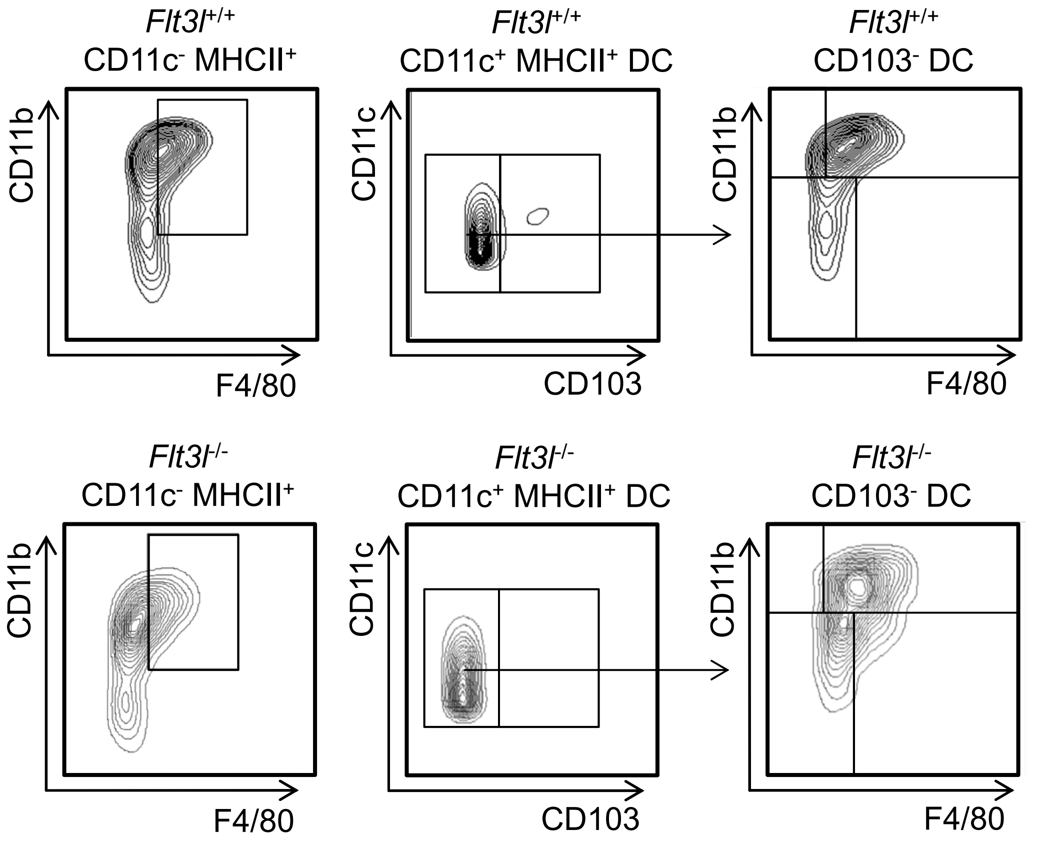

Supplemental Figure S4. DCs in the Flt3L-deficient mice.

Representative FACS plots for identification of DC subsets in healthy *Flt3l*$^{+/+}$ and *Flt3l*$^{-/-}$ mice, fed a normal chow. After exclusion of TCRβ^{+}/CD19^{+} T and B cells, macrophages were defined as CD11c^{-} MHCII^{+} CD11b^{+} F4/80^{+} (left panel), and CD11c^{+} MHCII^{+} DCs were further subdivided into CD103^{+} and CD103^{-} DCs (middle panel). CD103^{-} DCs were further subdivided into CD11b^{+} F4/80^{-}, CD11b^{+} F4/80^{+} and CD11b^{-}F4/80^{-} DCs (right panel).
